# Cancer Treatment Before and After Physician-Pharmacy Integration

**DOI:** 10.1001/jamanetworkopen.2024.12998

**Published:** 2024-05-23

**Authors:** Genevieve P. Kanter, Pelin Ozluk, Winnie Chi, Michael J. Fisch, David Debono, Ravi B. Parikh, Mireille Jacobson, Justin E. Bekelman, Andrea DeVries

**Affiliations:** 1 Department of Health Policy and Management, Sol Price School of Public Policy, University of Southern California, Los Angeles; 2 Leonard D. Schaeffer Center for Health Policy and Economics, University of Southern California, Los Angeles; 3 Elevance Health Inc, Indianapolis, Indiana; 4 Carelon Medical Benefits Management, Chicago, Illinois; 5 Department of Medical Ethics and Health Policy, Perelman School of Medicine, University of Pennsylvania, Philadelphia; 6 Penn Center for Cancer Care Innovation, Abramson Cancer Center, University of Pennsylvania, Philadelphia; 7 Corporal Michael J. Crescenz Veterans Affairs Medical Center, Philadelphia, Pennsylvania; 8 Leonard Davis School of Gerontology, University of Southern California, Los Angeles; 9 Department of Radiation Oncology, Perelman School of Medicine, University of Pennsylvania, Philadelphia

## Abstract

**Importance:**

Integration of pharmacies with physician practices, also known as medically integrated dispensing, is increasing in oncology. However, little is known about how this integration affects drug use, expenditures, medication adherence, or time to treatment initiation.

**Objective:**

To examine the association of physician-pharmacy integration with oral oncology drug expenditures, use, and patient-centered measures.

**Design, Setting, and Participants:**

This cohort study used claims data from a large commercial insurer in the US to analyze changes in outcome measures among patients treated by pharmacy-integrating vs nonintegrating community oncologists in 14 states between January 1, 2011, and December 31, 2019. Commercially insured patients were aged 18 to 64 years with 1 of the following advanced-stage diagnoses: breast cancer, colorectal cancer, kidney cancer, lung cancer, melanoma, or prostate cancer. Data analysis was conducted from May 2023 to March 2024.

**Exposure:**

Treatment by a pharmacy-integrating oncologist, ascertained by the presence of an on-site pharmacy or nonpharmacy dispensing site.

**Main Outcomes and Measures:**

Oral, intravenous (IV), total, and out-of-pocket drug expenditures for a 6-month episode of care; share of patients prescribed oral drugs; days’ supply of oral drugs; medication adherence measured by proportion of days covered; and time to treatment initiation. The association between an oncologist’s pharmacy integration and each outcome of interest was estimated using the difference-in-differences estimator.

**Results:**

Between 2012 and 2019, 3159 oncologists (745 females [27.1%], 2002 males [72.9%]) treated 23 968 patients (66.4% female; 53.4% aged 55-64 years). Of the 3159 oncologists, 578 (18.3%) worked in practices that integrated with pharmacies (with a low rate in 2011 of 0% and a high rate in 2019 of 31.5%). In the full sample (including all cancer sites), after physician-pharmacy integration, no significant changes were found in oral drug expenditures, IV drug expenditures, or total drug expenditures. There was, however, an increase in days’ supply of oral drugs (5.96 days; 95% CI, 0.64-11.28 days; *P* = .001). There were no significant changes in out-of-pocket expenditures, medication adherence, or time to treatment initiation of oral drugs. In the breast cancer sample, there was an increase in oral drug expenditures ($244; 95% CI, $41-$446; *P* = .02) and a decrease in IV drug expenditures (–$4187; 95% CI, –$8293 to –$80; *P* = .05).

**Conclusions and Relevance:**

Results of this cohort study indicated that the integration of oncology practices with pharmacies was not associated with significant changes in expenditures or clear patient-centered benefits.

## Key Points

**Question:**

How does physician-pharmacy integration change oral oncology drug expenditures, use, and patient-centered measures?

**Findings:**

In this cohort study of 3159 community oncologists of pharmacy-integrating physicians and nonintegrating physicians, there was a slight increase in use but no significant change in oral oncology drug expenditures after integration. Physician-pharmacy integration was associated with no discernible benefits for patients in out-of-pocket expenditures, medication adherence, or time to treatment initiation.

**Meaning:**

Findings of this study suggest that physician-pharmacy integration is not associated with change in the care of patients with cancer.

## Teaser

This cohort study examines the implications of physician-pharmacy integration for the use of, cost of, and adherence to anticancer drugs among patients with late-stage cancer.

## Introduction

An important emerging pattern in oncology is the integration of pharmacies with physician practices. Between 2010 and 2019, the share of oncologists in practices with an on-site pharmacy increased from 13% to 32%.^1^ This rapid increase has been observed among both community oncologists and hospital-based oncologists.

However, little is known about how physician-pharmacy integration (also known as medically integrated dispensing) is affecting patient care, drug use, and expenditures. Small-scale studies suggest that physician-pharmacy integration has been associated with improved patient experience and outcomes.^2^ Through improved patient monitoring and care coordination, physician-pharmacy integration might reduce time from diagnosis to start of treatment, avert severe toxic effects and drug interactions, and improve medication adherence. Pharmacy integration could also reduce unnecessary health care spending by incentivizing the use of oral therapies, which are dispensed by pharmacies, and integration could act as a counterweight against the overuse of expensive infused therapies.^3^ On-site pharmacies could also avert waste that can occur with mail orders, whose unused supplies must be discarded when a dose is changed or a course of treatment is discontinued.^4,5^

On the other hand, if physicians can financially benefit from both oral therapies dispensed at in-house pharmacies and intravenous (IV) therapies, pharmacy integration could lead to increased overall use of both oral and infused therapies as well as high expenditures. Integration could also incentivize the overuse of particularly profitable oral therapies. With the increasing number of oral oncology drugs in the pipeline and receiving approval each year,^6,7,8^ pharmacy integration could exacerbate financial toxicities associated with high out-of-pocket costs and nonadherence to oral anticancer drugs.

In this study, we aimed to examine the association of physician-pharmacy integration with oral oncology drug expenditures, use, and patient-centered measures. We analyzed the association between integration and oral, IV, and total (oral and IV) drug expenditures. We also examined the implications of integration for patients, analyzing changes in out-of-pocket expenditures, medication adherence, and time to treatment initiation.

## Methods

### Design and Population

We conducted a retrospective cohort study of practicing oncologists and commercially insured patients who were treated by these oncologists between January 1, 2011, and December 31, 2019. We tracked oncologists longitudinally through the study period. Patients were followed for 180 days after their initial diagnosis (ie, for a 6-month episode of care). Data were collected at the patient level, grouped according to the treating physician, and analyzed at the physician level. The University of Pennsylvania Institutional Review Board approved this study and waived the informed consent requirement because the research was considered to be minimal risk. We followed the Strengthening the Reporting of Observational Studies in Epidemiology (STROBE) reporting guideline.

The physician population consisted of community oncologists practicing in the US between 2011 and 2019 who treated commercially insured patients in the sample. Community oncologists are oncologists who own their own practice and are not part of a hospital or an academic or medical teaching institution.^9^ This group of oncologists experienced the most rapid increases in integration during the study period^1^ and likely derived the greatest financial benefit from pharmacy integration because of their direct ownership stakes in on-site pharmacies.

The patient population consisted of US patients aged 18 to 64 years who were treated by the oncologists in the sample between 2011 and 2019 and who participated in health plans offered by a large, national commercial insurer. Because integrated pharmacies could particularly affect treatment decisions on cancers for which there are both oral and IV therapies (because of potential substitution between oral and IV therapies), we focused on patients diagnosed with those cancers. Specifically, the sample consisted of individuals diagnosed with 1 of 6 cancers in their advanced or metastatic stage: breast cancer, colorectal cancer, kidney cancer, lung cancer, melanoma, or prostate cancer (eAppendix 1 in Supplement 1 provides the diagnostic codes).

We restricted the sample to individuals who were diagnosed between 2011 and 2019, underwent treatment between 2012 and 2019, and were newly diagnosed (ie, did not have claims associated with a cancer diagnosis in the preceding year). A cancer diagnosis was based on the presence of 2 or more diagnostic claims for cancer associated with an outpatient visit or 1 or more diagnostic claims for cancer associated with an emergency department visit or inpatient visit. The date of diagnosis was defined as the date of the first qualifying diagnostic claim. We excluded individuals with more than 1 cancer diagnosis during the study period.

### Data

Data on oncologists were obtained from the health information company IQVIA. IQVIA’s OneKey (previously SK&A) is an annual compilation of office-based physicians practicing in the US and is estimated to include 74% of oncologists billing Medicare fee-for-service and 90% of physicians across all specialties.^10,11,12^ As described elsewhere,^1^ we linked physician and practice data to annual data on pharmacies using organizational names and locations to identify oncologists and oncology practices that opened and operated on-site pharmacies. Annual data on pharmacies were obtained from the National Council for Prescription Drug Programs, a nonprofit organization that collects registration information on a wide range of pharmacies, including chain and independent retail pharmacies, clinic pharmacies, and nonpharmacy dispensing sites (eg, in-office sites).^13^

Data on patient-level use and expenditures were obtained from the Healthcare Integrated Research Environment (HIRE). HIRE is a repository of medical and pharmacy claims data for 78 million members managed by 14 commercial health plans. These plans cover a racially and ethnically diverse population across the US in the following states: California, Colorado, Connecticut, Georgia, Indiana, Kentucky, Maine, Missouri, Nevada, New Hampshire, New York, Ohio, Virginia, and Wisconsin. Patients were attributed to a primary oncologist each year based on physician share of medical and pharmacy claims (eAppendix 2 in Supplement 1).

### Outcomes and Covariates

Annual physician-level outcomes included mean (calculated among all patients treated by that physician) oral drug expenditures, IV drug expenditures, and total drug expenditures for a patient’s 6-month care episode beginning 7 days before the diagnosis date and ending 180 days after the diagnosis date. To mitigate bias from skewness and extreme outliers in the expenditure distributions, we winsorized expenditures at the physician level at the 99th percentile. In addition, we conducted robustness checks with log-transformed expenditures. Other primary outcomes included, at the physician level, mean per-patient-episode out-of-pocket expenditures, mean days’ supply of oral drugs, mean proportion of days covered (a measure of medication adherence), mean time from diagnosis to treatment initiation in days, and mean share of patients prescribed oral drugs.

Covariates, when included, were physician-level practice characteristics that varied over time: mean patient age, percentage of female patients, percentage of Black patients, mean household income of patient zip code areas, percentage of patients living in an urban area, mean Charlson Comorbidity Index, and percentage of patients who had been diagnosed with each of the cancers of interest. These socioeconomic and clinical characteristics have been associated with expenditures, medication adherence, and other outcomes of interest. The eAppendix 3 in Supplement 1 explains the treatment of time-invariant physician characteristics (eg, subspecialty).

### Statistical Analysis

To estimate the association between an oncologist’s integration with a pharmacy and each outcome of interest, we used a variant (introduced by Callaway and Sant’Anna^14^) of the difference-in-differences (DD) estimator.^15^ This estimator accounted for the staggered timing of pharmacy integration because practices adopted pharmacy integration in different years throughout the study period. The Callaway-Sant’Anna DD estimator was implemented using 2 steps.

First, the average treatment effect on the treated (ATT) parameter was estimated for each cohort *c* of physicians integrating with pharmacies, where *c* = 2012 was the group of physicians who integrated in the first year of the study (2011 was year 0, the preperiod), *c* = 2013 was the group of physicians who integrated in the following year, and so on. The parameter ATT(*c,t*) was calculated by comparing changes in mean patient outcomes for those treated by physicians in cohort *c* to changes in mean outcomes for patients treated by physicians who did not integrate. More specifically, for each integrating physician cohort *c*, the integration parameter was estimated for every postintegration year *t*, where *t* = 0 was the year of integration, *t* = 1 was the year after integration, and so on. The parameter ATT(*c*,*t*) was the mean association of pharmacy integration for integrating cohort *c* in postintegration year *t*, estimated using the standard 2 × 2 DD framework. This model specification is provided in eAppendix 3 in Supplement 1. Note that the DD estimator accounted for time-invariant characteristics of the physician and time-varying confounders with similar trends across control (nonintegrating) and treatment (pharmacy-integrating) groups.

Second, the overall estimated parameter for integration was produced by aggregating ATT(*c*,*t*) over all integrating physician cohorts *c* and postintegrating years *t*. Aggregation and clustering of SEs at the physician level, via multiplier bootstrapping, were conducted as previously described.^14^

The DD method required the assumption of parallel trends; namely, that trends among oncologists who did not integrate be parallel to those that would have been observed among integrating oncologists had they not integrated. Although this assumption could not be directly tested because it would have required knowing what would have been observed had integrating physicians not acquired an on-site pharmacy, we reported evidence in support of assumption validity in eAppendix 3 in Supplement 1. In addition to producing an overall estimate of the parameter for pharmacy integration, DD methods allowed us to examine how patterns of cancer care evolved in each year after integration. We reported these event studies and dynamic effects graphically, showing ATT(*c*,*t*) at each postintegration year *t*, aggregated over all integration cohorts.

We conducted a statistical analysis for all cancer sites combined (full sample) and for the breast cancer site (breast cancer sample), the site that accounted for the largest number of physicians (n = 2570) and for which we were sufficiently powered to detect plausible effect sizes. We also conducted an exploratory analysis of expenditure and use outcomes, stratifying by brand vs generic status of the oral drug as well as by therapeutic category (chemotherapy, hormone therapy, targeted therapy, and supportive care).

Data analysis was conducted from May 2023 to March 2024 using Stata 17 (StataCorp LLC) and the csdid module to implement the Callaway-Sant’Anna DD estimator.^16^ All hypothesis tests were 2-sided, with α = .05 indicating statistical significance.

## Results

In 2012, 44 (4.2%) of the 1043 community oncologists in the sample worked in practices with on-site pharmacies (eAppendix 4 in Supplement 1). By 2019, 261 (27.6%) of the 945 oncologists were in pharmacy-integrated practices. Between 2012 and 2019, a total of 3159 oncologists (745 females [27.1%], 2002 males [72.9%]) treated 23 968 patients (66.4% female and 33.6% male; 53.4% with mean age group of 55-64 years). Of the 3159 oncologists, 578 (18.3%) worked in pharmacy-integrated practices (with a low rate in 2011 of 0% and a high rate in 2019 of 31.5%). Table 1 reports a summary of physician and physician-level mean patient characteristics for the 2011 to 2019 sample. Oncologists with practices that integrated with pharmacies were mostly similar to those with practices that did not integrate. However, more pharmacy-integrating oncologists were in higher-volume practices: 305 (53.9%) were in practices with a daily patient volume greater than 90 patients compared with 769 nonintegrating oncologists (31.5%). The patient mix of pharmacy-integrating practices also consisted of a smaller share of Medicare beneficiaries compared with nonintegrating practices; 22.4% of nonintegrating practices treated more than 55 Medicare beneficiaries per 100 monthly site visitors compared with 29.8% of pharmacy-integrating practices.

**Table 1.** Physician and Patient Characteristics by Physician-Pharmacy Integration Status From 2011 to 2019

Characteristic	No. (%)		
	All	Nonintegrating	Pharmacy-integrating
**Physician characteristics**			
No. of physicians	3159	2581	578
Sex (n = 2747)			
Female	745 (27.1)	604 (27.1)	141 (27.4)
Male	2002 (72.9)	1628 (72.9)	374 (72.6)
Years since medical school graduation			
≤10	296 (9.4)	262 (10.1)	34 (5.9)
11-24	1212 (38.4)	950 (36.8)	262 (45.3)
25-39	1015 (32.1)	836 (32.4)	179 (31.0)
≥40	636 (20.1)	533 (20.7)	103 (17.8)
Subspecialty (n = 2747)			
Medical oncology	2647 (96.3)	2142 (96.0)	505 (98.1)
Gynecologic oncology	32 (1.2)	32 (1.4)	0
Other^a^	68 (2.5)	58 (2.6)	10 (1.9)
Urbanicity (n = 2747)			
Metropolitan	2611 (95.0)	2108 (94.4)	503 (97.7)
Micropolitan (suburban)	136 (5.0)	124 (5.6)	12 (2.3)
Site daily patient volume, No. (n = 3009)			
≤30	623 (20.7)	562 (23.0)	61 (10.8)
31-90	1311 (43.6)	1111 (45.5)	200 (35.3)
>90	1074 (35.7)	769 (31.5)	305 (53.9)
No. of Medicare beneficiaries (n = 1732)			
≤100	68 (3.9)	60 (5.0)	8 (1.5)
101-300	353 (20.4)	290 (24.2)	63 (11.8)
301-600	699 (40.4)	486 (40.5)	213 (40.1)
601-900	381 (22.0)	232 (19.3)	149 (28.0)
>901	231 (13.3)	132 (11.0)	99 (18.6)
No. of Medicare beneficiaries per 100 monthly site visitors (n = 1662)			
≤10	133 (8.0)	96 (8.5)	37 (7.0)
11-25	474 (28.5)	298 (26.2)	176 (33.5)
26-40	369 (22.2)	242 (21.3)	127 (24.1)
41-55	230 (13.8)	162 (14.2)	68 (12.9)
>56	456 (27.5)	338 (29.8)	118 (22.4)
**Physician-level patient characteristics**			
% Of female patients, mean (SD)	66.4 (42.3)	66.7 (42.4)	65.4 (41.6)
% Of male patients, mean (SD)	33.6 (42.3)	33.3 (42.4)	34.6 (41.6)
% Of patients with cancer diagnosis, mean (SD)			
Advanced breast cancer	42.9 (44.6)	43.3 (44.9)	41.1 (42.9)
Advanced lung cancer	24.4 (38.3)	24.3 (38.6)	24.9 (37.2)
Advanced colorectal cancer	21.1 (36.0)	21.2 (36.4)	20.8 (34.5)
Advanced prostate cancer	4.6 (19.3)	4.7 (19.4)	4.3 (18.6)
Advanced kidney cancer	3.5 (16.2)	3.5 (16.3)	3.9 (15.7)
Advanced melanoma	3.4 (16.3)	3.1 (15.7)	5.0 (18.6)
% Of patients in age group, mean (SD)			
18-24 y	0.1 (3.0)	0.1 (2.5)	0.3 (4.6)
25-39 y	6.3 (21.2)	6.1 (2.1)	7.2 (2.2)
40-54 y	40.2 (43.1)	40.8 (43.5)	37.3 (40.8)
55-64 y	53.4 (43.9)	53.0 (44.3)	55.2 (0.4)
Charlson Comorbidity Index, mean (SD)	8.8 (1.2)	8.8 (1.2)	8.8 (1.3)
Patient zip code characteristics, mean (SD)			
% Of Black patients	11.4 (15.7)	11.4 (15.8)	11.4 (15.2)
% Of patients living in urban area	92.8 (23.5)	92.9 (23.5)	92.3 (23.5)
Median household income, US$	69 673 (26 357)	70 160 (26 774)	67 550 (24 368)

^a^ Other subspecialty included radiation oncology and surgical oncology.

The mean share of patients diagnosed with advanced breast cancer was 42.9% (Table 1). The share of patients was 40.2% for those aged 40 to 54 years and 53.4% for those aged 55 to 64 years. There were few differences in the characteristics of patients treated by pharmacy-integrating vs nonintegrating oncologists.

In the full sample, we found no difference in the share of patients prescribed oral drugs between pharmacy-integrating oncologists and nonintegrating oncologists (Table 2). We did, however, observe an increase in the mean days’ supply of oral drugs (5.96 days; 95% CI, 0.64-11.28 days; *P* = .001). There were no significant differences in oral, IV, or total drug expenditures. We found similar nonsignificant results with covariates included (eAppendix 5 in Supplement 1) and using log-transformed expenditures (eAppendix 6 in Supplement 1).

**Table 2.** Change in 6-Month Episode Use and Expenditures After Physician-Pharmacy Integration^a^

Outcome	Estimate (95% CI)	*P* value	Baseline (range)^b^
**All cancer sites (full sample)**			
Use			
Mean share of patients prescribed oral drugs, percentage points	5.2 (0.0 to 10.6)	.06	35.0 (0 to 100)
Mean days’ supply of oral drugs	5.96 (0.64 to 11.28)	.001	20.51 (0 to 251)
Expenditures, US$			
Oral drug	351 (−287 to 991)	.28	1027 (0 to 14 105)
IV drug	−238 (−4579 to 4103)	.91	16 015 (0 to 224 107)
Total	1029 (−4088 to 6146)	.69	20 382 (0 to 272 395)
**Breast cancer site (breast cancer sample)**			
Use			
Mean share of patients prescribed oral drugs, percentage points	8.1 (−0.9 to 17.2)	.08	38.4 (0 to 100)
Mean days’ supply of oral drugs	7.31 (0.96 to 13.67)	.02	23.57 (0 to 251)
Expenditures, US$			
Oral drug	244 (41 to 446)	.02	354 (0 to 9239)
IV drug	−4187 (−8293 to −80)	.05	12 262 (0 to 258 680)
Total	−2894 (−7270 to 1481)	.20	14 725 (0 to 361 906)

Abbreviation: IV, intravenous.

^a^ The comparison group comprised patients who were treated by nonintegrating oncologists. The full sample included 3159 physicians and 9805 physician-year observations. The breast cancer sample included 2570 physicians and 4334 physician-year observations. Difference-in-differences estimates are of mean per-patient expenditures and use measures at the physician level (ie, averaged across patients) without covariates. Estimation was based on sample of patients who were diagnosed with late-stage breast cancer, colorectal cancer, lung cancer, melanoma, prostate cancer, and kidney cancer between 2010 and 2019 and who received treatment between 2011 and 2019. Calculations are based on a 6-month episode, beginning 7 days prior to the diagnosis date and ending 180 days after the diagnosis date. SEs are clustered at the physician level.

^b^ Baseline was measured in the year prior to pharmacy integration among physicians who integrated.

In the breast cancer sample (Table 2; eAppendix 5 in Supplement 1), we again found a significant increase in the days’ supply of oral drugs (7.31; 95% CI, 0.96-13.67; *P* = .02) but no change in the share of patients prescribed oral drugs. For this group of patients, we found an increase in oral drug expenditures of $244 (95% CI, $41-$446; *P* = .02) from a baseline mean of $354. There were also significant decreases in IV drug expenditures (–$4187; 95% CI, –$8293 to $80; *P* = .05) from a baseline mean of $12 262, but there was no significant difference in total drug expenditures.

We found no differences in out-of-pocket oral drug expenditures for patients treated by pharmacy-integrating oncologists compared with nonintegrating oncologists for either the full sample or the breast cancer sample (Table 3). There were also no differences in medication adherence (as measured by proportion of days covered) or in time to treatment initiation of oral drugs for either sample.

**Table 3.** Change in Patient Out-of-Pocket Expenditures, Medication Adherence, and Time to Treatment Initiation After Physician-Pharmacy Integration^a^

Outcome	Estimate (95% CI)	*P* value	Baseline (range)^b^
**All cancer sites (full sample)**			
Out-of-pocket expenditures for oral drugs, US$	4 (−28 to 36)	.82	38 (0 to 917)
Medication adherence measure: proportion of days covered	0.01 (−0.06 to 0.09)	.65	0.47 (0.01 to 1.00)
Time to treatment initiation, d	−5.83 (−20.93 to 9.28)	.47	53.28 (0.00 to 180.00)
**Breast cancer site (breast cancer sample)**			
Out-of-pocket expenditures for oral drugs, US$	−30 (−67 to 7)	.11	67 (0 to 3697)
Medication adherence measure: proportion of days covered	0.01 (−0.07 to 0.09)	.84	0.49 (0.02 to 1.00)
Time to treatment initiation, d	0.74 (−15.52 to 16.99)	.93	64.34 (0.00 to 180.00)

^a^ The comparison group comprised patients who were treated by nonintegrating oncologists. The full sample included 3159 physicians and 9805 physician-year observations. The breast cancer sample included 2570 physicians and 4334 physician-year observations. Difference-in-differences estimates are of mean per-patient expenditures and use measures at the physician level (ie, averaged across patients) without covariates. Estimation was based on a sample of patients who were diagnosed with late-stage breast cancer, colorectal cancer, lung cancer, melanoma, prostate cancer, and kidney cancer between 2010 and 2019 and who received treatment between 2011 and 2019. Calculations are based on a 6-month episode, beginning 7 days prior to the diagnosis date and ending 180 days after the diagnosis date. SEs are clustered at the physician level.

^b^ Baseline was measured in the year prior to pharmacy integration among physicians who integrated.

Using event study plots, we examined the trajectory of changes in the outcomes of interest (Figure; eAppendix 7 in Supplement 1). The point estimate for the share of patients prescribed an oral drug (Figure, A) increased more for integrating oncologists than for nonintegrating oncologists. Furthermore, this gap continued to increase in the years after physician-pharmacy integration. (The flat blue line prior to *t* = 0 indicates no difference in outcomes between patients of pharmacy-integrating oncologists and those of nonintegrating oncologists in the years before integration.) A similar pattern was seen with mean days’ supply of oral drugs (Figure, B). The point estimate for the mean time to treatment initiation decreased more for patients treated by pharmacy-integrating oncologists and continued to decrease with each successive year (Figure, D). These 3 event study plots show a graphical gap opening between patients treated by pharmacy-integrating oncologists and those treated by nonintegrating oncologists.

**Figure.**
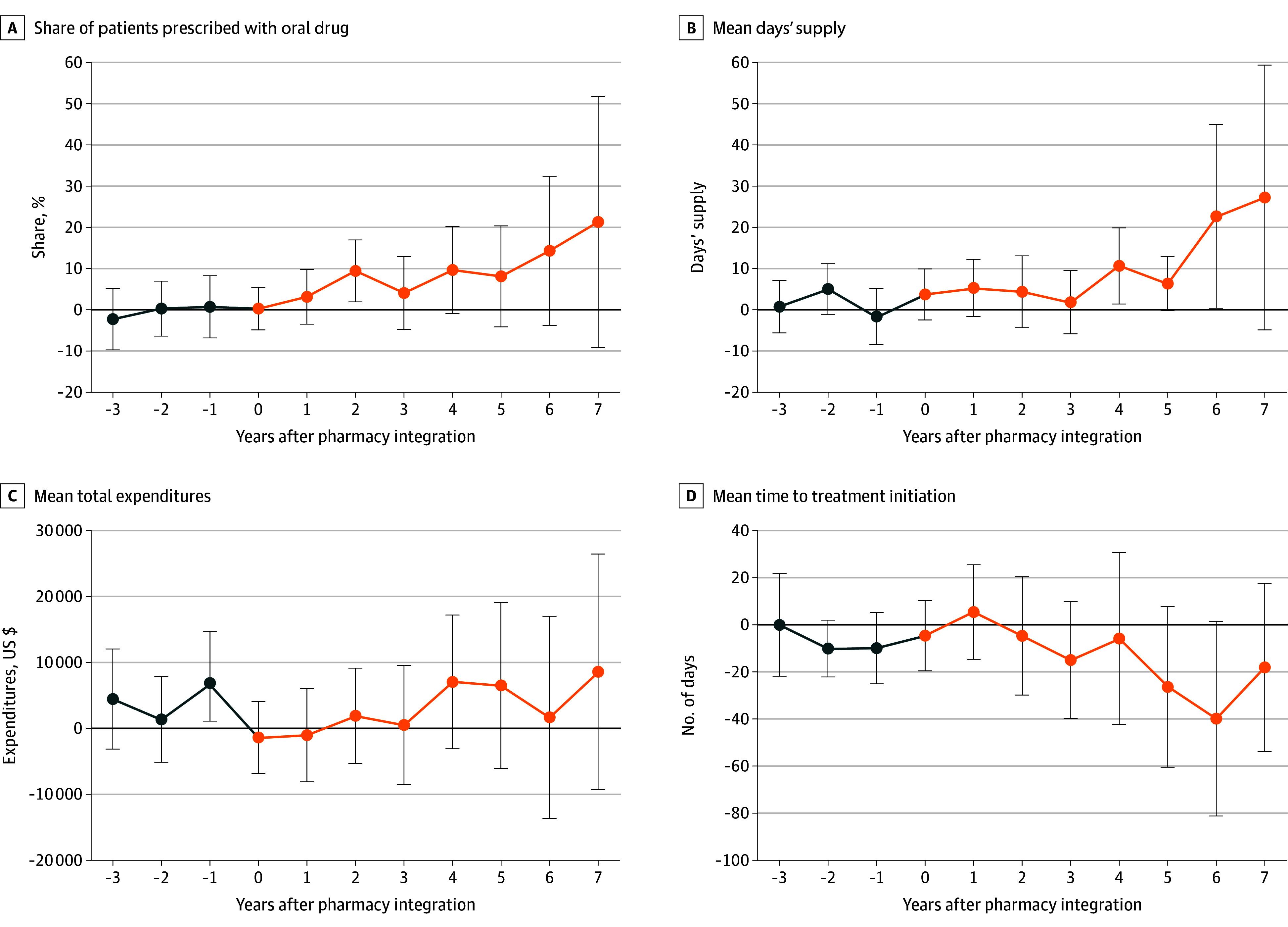
Event Studies Error bars represent 95% CIs; black line represents difference in outcome between pharmacy-integrating physicians and nonintegrating physicians before integration; and orange line represents difference in outcome between pharmacy-integrating physicians and nonintegrating physicians after integration. Total expenditures include oral and intravenous drugs.

By contrast, no divergence was observed in oral, IV, total, or out-of-pocket drug expenditures (Figure, C; eAppendix 7 in Supplement 1). Similarly, there was no gap in mean proportion of days covered (eAppendix 7 in Supplement 1).

In exploratory analyses, we found no difference between pharmacy-integrating and nonintegrating oncologists in expenditures for either branded or generic oral drugs (eAppendix 8 in Supplement 1). However, we did observe significant increases in the share of patients prescribed oral chemotherapy (2.4 percentage points; 95% CI, 0.7-4.1 percentage points; *P* = .006) and in the share of patients prescribed oral hormone therapy (4.5 percentage points; 95% CI, 1.4-7.6 percentage points; *P* = .005). There were no changes in the share of patients prescribed oral targeted therapy or oral supportive care (eAppendix 8 in Supplement 1).

## Discussion

In this retrospective cohort study of physician-pharmacy integration, we found that, after integration, there was an increase in oral drug use, increasing the days’ supply of oral drugs prescribed by about 6 days. However, there was no associated increase in the share of patients who were prescribed oral therapies.

Among patients with breast cancer, there was a 69% increase ($244 divided by $354 [baseline]) in oral drug expenditures. There was concurrently a 34% decrease ($4187 divided by $12 262 [baseline]) in IV drug expenditures, but on net, there was no significant change in total expenditures. This shift toward oral expenditures and away from IV expenditures was not observed in the full sample of all cancers, pointing to heterogeneity in substitution between oral and IV therapies across different cancer types after integration.

For patients, physician-pharmacy integration was not associated with increases in out-of-pocket payments or medication adherence rates. Event study plots showed a decrease in time to treatment initiation in each successive year after an oncologist practice opened an on-site pharmacy, but the overall decrease of 6 days was not statistically significant and likely not clinically significant. There may well be a limit to how much physician-pharmacy integration can reduce time to treatment initiation since many oral anticancer drugs require prior authorization,^17^ which is believed to delay the treatment start date by 2 weeks on average.^18^

Small-scale studies have suggested that physician-pharmacy integration is associated with reduced waste.^4^ With an on-site pharmacy, physicians can, in principle, switch therapies more quickly if adverse events arise because they can prescribe and dispense therapies for fewer days, thereby preventing waste. If this were the case, however, we should have observed a decrease in mean days’ supply among pharmacy-integrated practices as physicians prescribed shorter courses. Instead, we observed an increase in mean days’ supply. Since reimbursements increase with each additional pill, physicians may have been responding more to the additional revenues from increasing days’ supply than to the additional prescribing flexibility permitted by on-site pharmacies.

Overall, these findings suggest that, although physician-pharmacy integration has not resulted in large expenditure increases, it has not yielded large gains for patients or practices either. As shown in other experiences of vertical integration in health care, organizational integration may lead to financial integration but not necessarily clinical integration.^19^

### Limitations

There were several limitations to this study. Because the analysis relied on claims data, we were not able to evaluate patient experience or use patient-reported outcomes to assess adherence. We did not take into account practice participation in the Centers for Medicare & Medicaid Services Oncology Care Model,^20^ which was implemented in 2016 and may have been more likely to attract larger practices (which, in turn, were more likely to have on-site pharmacies). We were also unable to include indicators for participation in group purchasing organizations and oncology practice networks, which can affect drug acquisition costs of specific products because historical data on participation were not available. In addition, although we were able to analyze whether an oncologist at a pharmacy-integrating practice prescribed a specific drug, we were not able to analyze whether that drug was dispensed at that practice’s pharmacy. The sample was limited to the experience of a single commercial insurer with a presence in states in which physician-pharmacy integration was slightly slower than the national mean; thus, the findings may not be generalizable to markets with faster pharmacy integration. Additionally, the study focused on the 2011-2019 period and may not reflect current experience with physician-pharmacy integration, including any changes precipitated by the COVID-19 pandemic.

## Conclusions

This cohort study of commercially insured individuals treated at community oncology practices found increased oral drug use but no change in drug expenditures; there were also no increases in out-of-pocket payments or medication adherence rates. An important next step is to examine how hospital-based oncology practices, which have also rapidly increased the adoption of on-site dispensing, have adapted to pharmacy integration. These practices tend to be larger, have a different case and patient mix, and may face a different set of incentives, such as 340B Drug Pricing Program discounts, that may affect their practice patterns in ways that diverge from community practices. In addition, the implications of physician-pharmacy integration may be different for patients with Medicare insurance as well as marginalized racial and ethnic minority groups and populations with lower socioeconomic status. Although there appears to be no regulatory restraints required at the moment, study findings of heterogeneous responses and emerging gaps in use underscore the importance of continued monitoring of physician-pharmacy integration.

## Notes

Supplement 1. **eAppendix 1.** Diagnostic Codes for Sample Inclusion **eAppendix 2.** Patient Attribution to Physicians and Oral Drugs in Sample **eAppendix 3.** Difference-in-Differences Model Specification and Tests of Parallel Trends Assumption **eAppendix 4.** Annual Tabulation of Physicians and Patients **eAppendix 5.** Change in Utilization and Expenditures Following Pharmacy Integration, Difference-in-Differences Estimates With Covariates **eAppendix 6.** Change in Log-Transformed Expenditures Following Pharmacy Integration **eAppendix 7.** Event Studies of Oral Drug Expenditures, IV Drug Expenditures, Out-of-Pocket Expenditures, and Proportion of Days Covered **eAppendix 8.** Exploratory Analyses
eReferences

Supplement 2.
Data Sharing Statement
