# Supplementary material for: Cancer Treatment Before and After Physician-Pharmacy Integration
Source: JAMA Netw Open. 2024 May 23;7(5):e2412998. doi: 10.1001/jamanetworkopen.2024.12998 (PMC11117080; doi:10.1001/jamanetworkopen.2024.12998)
Supplement: Supplement 1. — eAppendix 1. Diagnostic Codes for Sample Inclusion eAppendix 2. Patient Attribution to Physicians and Oral Drugs in Sample eAppendix 3. Difference-in-Differences Model Specification and Tests of Parallel Trends Assumption eAppendix 4. Annual Tabulation of Physicians and Patients eAppendix 5. Change in Utilization and Expenditures Following Pharmacy Integration, Difference-in-Differences Estimates With Covariates eAppendix 6. Change in Log-Transformed Expenditures Following Pharmacy Integration eAppendix 7. Event Studies of Oral Drug Expenditures, IV Drug Expenditures, Out-of-Pocket Expenditures, and Proportion of Days Covered eAppendix 8. Exploratory Analyses eReferences [file jamanetwopen-e2412998-s001.pdf]

Supplemental Online Content

Kanter GP, Ozluk P, Chi W, et al. Cancer treatment before and after physician-pharmacy integration. *JAMA Netw Open*. 2024;7(5):e2412998.
doi:10.1001/jamanetworkopen.2024.12998

eAppendix 1. Diagnostic Codes for Sample Inclusion
eAppendix 2. Patient Attribution to Physicians and Oral Drugs in Sample
eAppendix 3. Difference-in-Differences Model Specification and Tests of Parallel Trends Assumption
eAppendix 4. Annual Tabulation of Physicians and Patients
eAppendix 5. Change in Utilization and Expenditures Following Pharmacy Integration, Difference-in-Differences Estimates With Covariates
eAppendix 6. Change in Log-Transformed Expenditures Following Pharmacy Integration
eAppendix 7. Event Studies of Oral Drug Expenditures, IV Drug Expenditures, Out-of-Pocket Expenditures, and Proportion of Days Covered
eAppendix 8. Exploratory Analyses
eReferences

This supplemental material has been provided by the authors to give readers additional information about their work.

© 2024 Kanter GP et al. *JAMA Network Open*

eAppendix 1. Diagnostic codes for sample inclusion

We used the standard protocol developed and regularly used by the commercial insurer to identify patients diagnosed with advanced stage cancers. A cancer diagnosis was based on the presence of two or more diagnostic claims for cancer associated with an outpatient visit with 2 claims at least 30 days apart, or one or more diagnostic claims for cancer associated with an emergency department visit or inpatient visit. The ICD-9 and ICD-10 codes for the malignances of interest—breast cancer, colorectal cancer, melanoma, lung cancer, prostate cancer, renal cancer--are listed below. The advanced stage of these cancers was identified by the presence of a diagnosis for a metastasis at any point during the study period given by the codes below. We excluded individuals who had claims associated with a prior cancer diagnosis in the preceding year or had been diagnosed with more than one cancer during the study period. Similar algorithms have been found to have good sensitivity and positive predictive value in commercial claims.[1]

Malignancies	ICD-9	ICD-10
Breast cancer	174.x, 175.x	C50.x
Colorectal cancer	153.x, 154.x	C18.x, C19.x
Melanoma	172.x	C43.x
Lung cancer	162.x	C34.x
Prostate cancer	185	C61
Renal cancer	189.0, 189.1	C64.1, C64.2, C64.9;<br>C65.1, C65.2, C65.9
Metastasis	197.x, 198.x, 199.0	C77.x, C79.x, C80.0x

© 2024 Kanter GP et al. *JAMA Network Open*

eAppendix 2. Patient attribution to physicians and oral drugs in sample

Patients were attributed to a primary oncologist each year based on physician share of medical and pharmacy claims. Because of our focus on oral drug prescribing, we attributed patients to a primary oncologist based on, first, who prescribed a plurality of a patient's pharmacy claims for 41 common oral anticancer drugs (see Table A2a below). If there were no oral therapy claims, we attributed patients to the oncologist associated with a plurality of medical claims for physician-administered anticancer therapies and oncology-related Evaluation and Management visits. Additional protocol details are described in Kanter et al.[2]

Table A2a. Oral anticancer medications (n=41)

Generic Name	Trade Name(s)	Disease(s)
Abemaciclib	Verzenio	breast
Abiraterone acetate	Yonsa, Zytiga	prostate
Afatinib	Gilotrif	lung
Alectinib	Alecensa	lung
Anastrozole	Arimidex	breast
Apalutamide	Erleada	prostate
Axitinib	Inlyta	renal
Bicalutamide	Casodex	prostate
Binimetinib	Mektovi	melanoma
Brigatinib	Alunbrig	lung
Cabozantinib	Cabometyx, Cometriq	renal
Capecitabine	Xeloda	breast, colorectal
Ceritinib	Zykadia	lung
Cobimetinib	Cotellic	melanoma
Crizotinib	Xalkori	lung
Dabrafenib	Tafinlar	lung, melanoma
Encorafenib	Braftovi	colorectal, melanoma
Enzalutamide	Xtandi	prostate
Erlotinib	Tarceva	lung
Everolimus	Afinitor, Zortress	breast, renal
Exemestane	Aromasin	breast
Gefitinib	Iressa	lung
Lapatinib	Tykerb	breast
Larotrectinib	Vitrakvi	lung, breast
Lenvatinib	Lenvima	renal
Letrozole	Femara	breast
Lorlatinib	Lorbrena	lung
Neratinib	Nerlynx	breast
Niraparib	Zejula	breast, prostate
Olaparib	Lynparza	breast, prostate
Osimertinib	Tagrisso	lung
Palbociclib	Ibrance	breast
Pazopanib	Votrient	renal
Regorafenib	Stivarga	colorectal
Ribociclib	Kisqali	prostate
Rucaparib	Rubraca	breast, prostate
Sorafenib	Nexavar	renal

© 2024 Kanter GP et al. *JAMA Network Open*

Sunitinib	Sutent	renal
Tamoxifen	Soltamox	breast
Trametinib	Mekinist	lung, melanoma
Vemurafenib	Zelboraf	melanoma

Table A2b. Oral supportive care medications (n=11)

Generic Name	Brand Name(s)	Route of Administration
Aprepitant	Emend	oral
Dexamethasone		oral
Dolasetron	Anzemet	oral
Granisetron	Kytril	oral
Granisetron	Sancuso	transdermal
Metoclopramide	Metozolv, Reglan	oral
Netupitant & Palonosetron	Akynzeo	oral
Ondansetron	Zofran	oral
Palonosetron	Aloxi	oral
Prochlorperazine	Compro	oral
Rolapitant	Varubi	oral

© 2024 Kanter GP et al. *JAMA Network Open*

eAppendix 3. Difference-in-differences model specification and tests of parallel trends assumption

Model specification

The model used to estimate the difference-in-differences (DD) parameter for cohort *c* and in post-integration year *t* was:

$$Y_{icy,t} = \alpha + \beta\, \mathbb{1}[Integ]_i + \gamma^{ct}\, \mathbb{1}[y > c]_{cy} \cdot \mathbb{1}[y - c = t]_{cyt} + \delta^{ct}\mathbb{1}[Integ]_i \cdot\, \mathbb{1}[y > c]_{cy} \cdot \mathbb{1}[y - c = t]_{cyt} + X_{iy}\Phi + \epsilon_{iyct}$$

where:

- $Y_{icy,t}$ is the outcome for physician *i* in cohort *c* in year *y* at *t* years pre/post-integration;
- $\mathbb{1}[Integ]_i$ is a binary variable indicating whether the physician *i* is an integrating physician;
- $\mathbb{1}[y \geq c]_{cy}$ is a binary variable indicating whether the year *y* is at or after the year of integration for integrating cohort *c*;
- $\mathbb{1}[y - c = t]_{cyt}$ is a variable that assumes the value 1 in any year *y* that is t years pre/post-integration for cohort c, and 0 otherwise; and
- X_{iy} is a vector of characteristics of physician i in year y.

The coefficient δ^{ct} identifies the DD parameter for average treatment-on-the-treated for cohort *c* at *t* years post-integration, ATT(*c*,*t*). This specification reflects the standard 2x2 DD setup, where the comparison group was the set of physicians who had not (yet) integrated with pharmacies by year *y*.

To obtain an estimate of the overall effect of pharmacy integration, ATT(*c*,*t*) estimates were aggregated over all integrating cohorts *c* and post-integration years *t*, as described in Callaway and Sant'Anna.[2] Standard errors were clustered at the physician level to account for autocorrelated errors associated with observing the same physician across multiple time periods. Aggregation of ATT(*c,t*) and clustering of standard errors via multiplier bootstrapping were conducted as described in Callaway and Sant'Anna.

Tests of parallel trends assumption

To assess the plausibility of the parallel trends assumption, we applied the test of parallel trends described in Callaway and Sant'Anna. For our analysis, the null hypothesis was no difference in trends in the 3 years prior to physician-pharmacy integration between practices that integrated and practices that did not. Note that this test is conservative and over-rejects the null hypothesis of no parallel trends.

We conducted tests for unconditional parallel trends--the assumption required for the DD analysis without covariates--and tests for conditional parallel trends--the assumption required for the DD analysis with covariates. Note that it is unlikely for both assumptions to simultaneously be true. If unconditional parallel trends holds—i.e., if there were no difference in the pre-period trends between integrating and non-integrating oncologists—then conditional parallel trends—i.e., parallel pre-period trends conditional on covariates—is unlikely to hold unless the covariates themselves are trending in the same way (even more unlikely). Also note that if parallel trends in levels of expenditures holds, then it is unlikely that parallel trends in log-transformed expenditures also holds, although the test may not be sufficiently powered to reveal that difference.

© 2024 Kanter GP et al. *JAMA Network Open*

For each outcome of interest, the X^2 statistic for the test and its associated p-value are reported below. For most outcome measures for the aggregated cancer site sample (full sample), we could not reject the null hypothesis that there was no difference in trends between integrating practices and non-integrating practices in the years prior to integration. Log-transformed expenditure outcomes were closer to the rejection threshold than level expenditures outcomes.

The null hypothesis of no difference in pre-integration trends was rejected more frequently for outcomes associated with the breast cancer sample and for tests of the conditional parallel trends assumption, i.e., the assumption that trends in outcomes are parallel conditional on the covariates.

Unconditional parallel trends tests

Outcome measures	X^2 statistic	p-value
**All cancer sites**		
Utilization		
Mean share of patients prescribed oral drugs	22.42	0.21
Mean days' supply of oral drugs	22.59	0.21
Expenditures		
Oral drug expenditures	19.85	0.34
IV drug expenditures	12.50	0.82
Total (oral + IV) expenditures	13.62	0.75
Log(oral drug expenditures)	26.01	0.10
Log(IV drug expenditures)	25.60	0.11
Log(total (oral + IV) expenditures)	29.14	0.05
Patient-centered measures		
Out-of-pocket expenditures on oral drugs	26.09	0.10
Medication adherence: proportion of days covered	293.08	<0.001
Time-to-treatment initiation	35.87	0.002
**Breast cancer**		
Utilization		
Mean share of patients prescribed oral drugs	38.03	0.002
Mean days' supply of oral drugs	18.46	0.360
Expenditures		
Oral drug expenditures	48.80	<0.001
IV drug expenditures	33.07	0.01
Total (oral + IV) expenditures	50.24	<0.001
Patient-centered measures		
Out-of-pocket expenditures on oral drugs	26.78	0.06
Medication adherence: proportion of days covered	2716.97	<0.001
Time-to-treatment initiation	394.98	<0.001

Conditional parallel trends tests

Outcome measures	X^2 statistic	p-value
**All cancer sites**		
Utilization		
Mean share of patients prescribed oral drugs	20.06	0.09
Mean days' supply of oral drugs	17.21	0.19

© 2024 Kanter GP et al. *JAMA Network Open*

Expenditures		
Oral drug expenditures	22.79	0.04
IV drug expenditures	18.74	0.13
Total (oral + IV) expenditures	20.52	0.08
Patient-centered measures		
Out-of-pocket expenditures on oral drugs	9.24	0.75
Medication adherence: proportion of days covered	400.03	<0.001
Time-to-treatment initiation	165.61	<0.001
**Breast cancer**		
Utilization		
Mean share of patients prescribed oral drugs	61.87	<0.001
Mean days' supply of oral drugs	12.61	0.76
Expenditures		
Oral drug expenditures	16.04	0.52
IV drug expenditures	89.84	<0.001
Total (oral + IV) expenditures	66.05	<0.001
Patient-centered measures		
Out-of-pocket expenditures on oral drugs	26.96	0.06
Medication adherence: proportion of days covered	249.14	<0.001
Time-to-treatment initiation	92.92	<0.001

© 2024 Kanter GP et al. *JAMA Network Open*

eAppendix 4. Annual tabulation of physicians and patients

	2011	2012	2013	2014	2015	2016	2017	2018	2019	Total
**Pharmacy integrated practices**										
No. of physicians	0	44	114	133	188	238	236	248	261	578
No. of patients	0	108	287	398	566	717	645	612	634	3,967
**Non-integrated practices**										
No. of physicians	1,282	999	1,110	1,046	908	858	764	692	684	2,581
No. of patients	3,035	2,343	2,815	2,700	2,260	2,048	1,906	1,445	1,449	20,001
**Total**										
No. of physicians	1,282	1,043	1,224	1,179	1,096	1,096	1,000	940	945	3,159
No. of patients	3,035	2,451	3,102	3,098	2,826	2,765	2,551	2,057	2,083	23,968

Note that the number of physicians in the Total column is the number of unique physicians in the full sample. This total is not the sum of the annual number of physicians because different physicians enter and exit the sample in different years throughout the sample period.

© 2024 Kanter GP et al. *JAMA Network Open*

eAppendix 5. Change in utilization and expenditures following pharmacy integration, difference-in-differences estimates with covariates

Outcome	Estimate (95% CI)	Baseline[1] (min,max)
**All cancer sites**		
Utilization		
Mean share of patients prescribed oral drugs	4.7% points (-1.4%,10.8%) p=0.13	35.0% (0%,100%)
Mean days' supply of oral drugs	3.39 (-3.25,10.03) p=0.32	20.51 (0,251)
Expenditures		
Oral drug expenditures	$318 (-$490,$1,128) p=0.13	$1,027 ($0,$14,105)
IV drug expenditures	$1,596 (-$3,884,$7,075) p=0.57	$16,015 ($0,$224,107)
Total expenditures	$2,693 (-$4,228,$9,614) p=0.45	$20,382 ($0,$272,395)
Patient-centered measures		
Out-of-pocket expenditures for oral drugs	-$18 (-$60,$24) p=0.40	$38 ($0,$917)
Medication adherence: proportion of days covered	-0.31 (-0.11,0.05) p=0.45	0.47 (0.01,1.00)
Time-to-treatment initiation (days)	0.77 (-22.36,23.90)	53.28 (0.00,180.00)
**Breast cancer**		
Utilization		
Mean share of patients prescribed oral drugs	9.7% (-1.1%,20.5%) p=0.08	38.4% (0%,100%)
Mean days' supply of oral drugs	6.00 (-1.83,13.83) p=0.13	23.57 (0,251)
Expenditures		
Oral drug expenditures	$304** ($12,$596) p=0.04	$354 ($0,$9,239)
IV drug expenditures	-$4,405** (-$8,483,-$326) p=0.03	$12,262 ($0,$258,680)
Total expenditures	-$2,862	$14,725

© 2024 Kanter GP et al. *JAMA Network Open*

	(-$8,665,$2,941)<br>p=0.33	($0,$361,906)
Patient-centered measures		
Out-of-pocket expenditures for oral drugs	-$34<br>(-$93,$24)<br>p=0.24	$67<br>($0,$3,697)
Medication adherence: proportion of days covered	-0.03<br>(-0.12,0.07)<br>p=0.56	0.49<br>(0.02,1.00)
Time-to-treatment initiation (days)	7.58<br>(-14.09,29.24)<br>p=0.49	64.34<br>(0.00,180.00)

* p<0.10 ** p<0.05 ***p<0.01

© 2024 Kanter GP et al. *JAMA Network Open*

eAppendix 6. Change in log-transformed expenditures following pharmacy integration

Outcome	Estimate (95% CI)	Baseline[1] (min,max)
Expenditures		
Oral drug expenditures	0.51 (-0.04,1.06) p=0.07	2.74 (0,9.55)
IV drug expenditures	0.13 (-0.48,0.73) p=0.68	7.04 (0,12.32)
Total expenditures	0.28 (-0.22,0.77) p=0.27	7.96 (0,12.52)

* p<0.10 ** p<0.05 ***p<0.01

© 2024 Kanter GP et al. *JAMA Network Open*

eAppendix 7. Event studies of oral drug expenditures, IV drug expenditures, out-of-pocket expenditures, and proportion of days covered

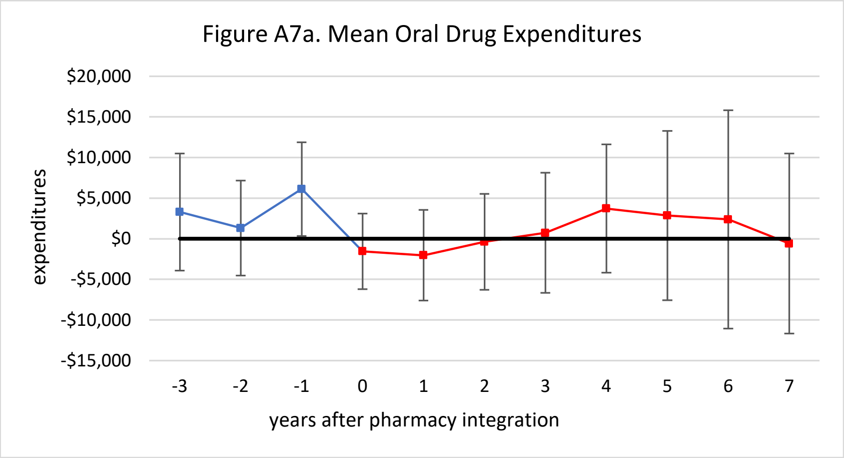

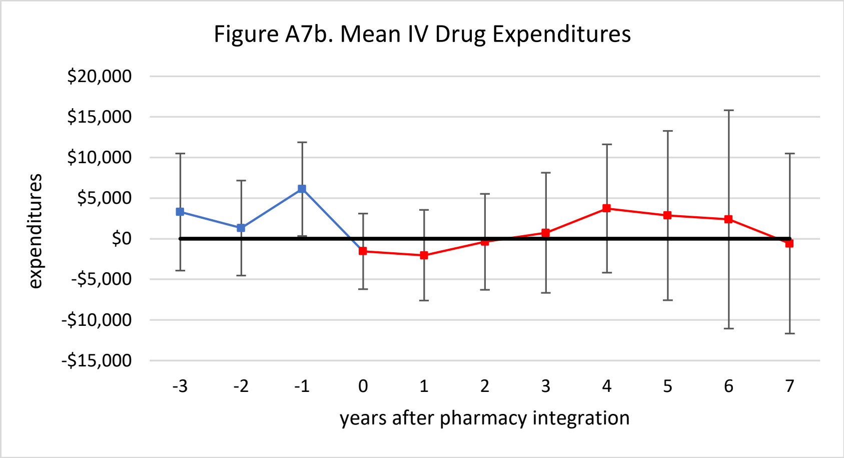

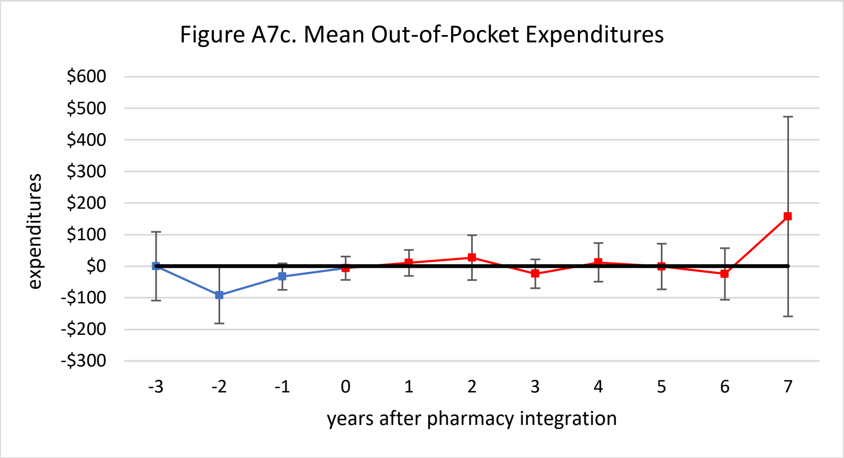

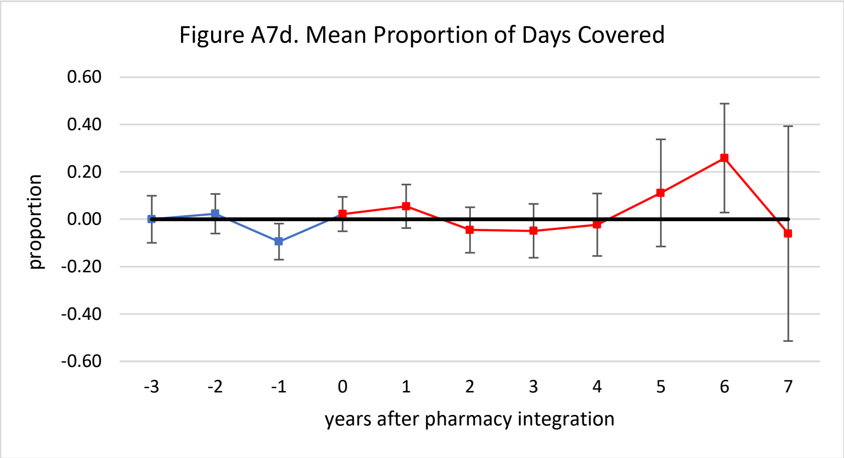

Note: Point estimates with 95% CI bars for outcomes in each year indexed to year of integration (t=0). Blue markers and lines show pre-integration years and red markers and lines show post-integration years. Deviations from x-axis at 0 reflect differences between integrating oncologists and non-integrating oncologists.

© 2024 Kanter GP et al. *JAMA Network Open*

eAppendix 8. Exploratory analyses

Change in expenditures by brand name vs. generic status of oral drug

Outcome	Estimate (95% CI)	Baseline[1] (min,max)
Expenditures		
Brand name oral drug expenditures	\$176 (-\$139,\$491) p=0.27	\$732 (\$0,\$9,479)
Generic oral drug expenditures	\$10 (-\$5,\$20) p=0.06	\$49 (\$0,\$595)

* p<0.10 ** p<0.05 ***p<0.01

Change in expenditures and share of patients prescribed oral drugs, by therapeutic category: oral chemotherapy, oral hormone therapy, oral targeted therapy, oral supportive care

Outcome	Estimate (95% CI)	Baseline[1] (min,max)
Expenditures		
Oral chemotherapy expenditures	\$132 (-\$15,\$280) p=0.08	\$205 (\$0,\$10,137)
Oral hormone therapy expenditures	\$287** (\$70,\$504) p=0.01	\$205 (\$0,\$3,584)
Oral targeted therapy expenditures	-\$68 (-\$794,\$657) p=0.85	\$1,329 (\$0,\$39,814)
Oral supportive care expenditures	-\$7 (-\$62,\$48) p=0.80	\$67 (\$0,\$5,499)
Share of patients		
Share of patients prescribed oral chemotherapy	2.4% pts*** (0.7%,4.1%) p=0.006	3.0% (0%,100%)
Share of patients prescribed oral hormone therapy	4.5% pts*** (1.4%,7.6%) p=0.005	8.7% (0%,100%)
Share of patients prescribed oral targeted therapy	-0.6% pts (-3.2%,2.0%) p=0.67	5.4% (0%,100%)
Share of patients prescribed oral supportive care	-1.1% pts (-6.3%,4.2%) p=0.69	21.2% (0%,100%)

* p<0.10 ** p<0.05 ***p<0.01

© 2024 Kanter GP et al. *JAMA Network Open*

eReferences

1. Whyte JL, Engel-Nitz NM, Teitelbaum A, Rey GG, Kallich JD. An evaluation of algorithms for identifying metastatic breast, lung, or colorectal cancer in administrative claims data. Med Care 2015; 53:e49-e57.
2. Kanter GP, Parikh RB, Fisch MJ, et al. Trends in medically integrated dispensing among oncology practices. JCO Oncol Pract 2022; 18:e1672-e1682.
3. Callaway B, Sant'Anna PHC. Difference-in-differences with multiple time periods. J Econom 2021; 225:200-230.

© 2024 Kanter GP et al. *JAMA Network Open*
