# Supplementary material for: Cancer Treatment Before and After Physician-Pharmacy Integration
Source: JAMA Netw Open. 2024 May 23;7(5):e2412998. doi: 10.1001/jamanetworkopen.2024.12998 (PMC11117080; doi:10.1001/jamanetworkopen.2024.12998)
Supplement: Supplement 2. — Data Sharing Statement [file jamanetwopen-e2412998-s002.pdf]

Data Sharing Statement

Kanter. Cancer Treatment Before and After Physician-Pharmacy Integration. *JAMA Netw Open*. Published May 23, 2024. doi:10.1001/jamanetworkopen.2024.12998

Data
Data available: No
